# A Case of Stanford Type A Aortic Dissection Complaining Only of Headache With a History of Migraine

**DOI:** 10.7759/cureus.20716

**Published:** 2021-12-26

**Authors:** Takuma Aoki, Mitsuhito Soh, Toru Hifumi, Norio Otani

**Affiliations:** 1 Department of Emergency and Critical Care Medicine, St. Luke’s International Hospital, Tokyo, JPN; 2 Department of Emergency Medicine and Critical Care, Kawasaki Municipal Kawasaki Hospital, Kanagawa, JPN

**Keywords:** emergency medicine, marfan syndrome, headache, aortic dissection, migraine

## Abstract

Stanford type A aortic dissection (SAAD) is a fatal condition in which patients often present with severe chest or back pain that radiates along the direction of propagation. In this report, we present the first published case of a patient complaining of migraine with aura as an initial manifestation of SAAD without the typical chest pain, back pain, or neurologic deficits. A 35-year-old, tall, male, night-shift taxi driver with a history of migraines arrived at the emergency department complaining of a slow-onset frontal headache that he attributed to migraine. Intravenous acetaminophen administration with fluid infusion did not improve his symptoms. An electrocardiogram showed ST depressions and a transthoracic echocardiogram showed severe aortic regurgitation with an aortic flap. He was diagnosed with Marfan syndrome complicated by SAAD and underwent surgical aortic root replacement. Aortic dissection may have a variety of initial manifestations; cardiovascular workup should be considered for migraine patients, especially those with Marfan-like features.

## Introduction

Migraine is one of the most commonly observed complaints in the emergency department [1-2]. Migraines are associated with cardiovascular diseases, such as patent foramen ovale, cerebrovascular infarction, and vasospastic angina [3-6], but they are not typically recognized as a prodrome of Stanford type A aortic dissection (SAAD) [7-8]. Previously, three cases of SAAD with a history of migraine have been reported [9-11]. Unlike previous reports, this case is novel in that it lacks typical pain features and neurologic deficits. Here, we share our experience of a challenging migraine-mimic aortic dissection that was successfully diagnosed using a transthoracic echocardiogram.

## Case presentation

**Figure 1:**
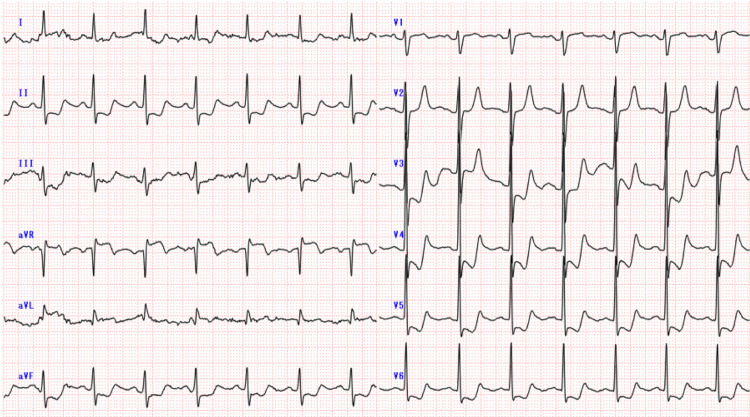
Electrocardiogram on admission

A 35-year-old Japanese male was brought to the emergency department for frontal headache. He had a history of migraine without cardiovascular disease or risk factors. He noted that his headache was associated with a right-sided visual disturbance that spontaneously resolved, which was typical for his usual migraines. The headache did not respond to over-the-counter painkillers and affected his ability to perform his job. The pain was characterized as gradual-onset and pulsatile, lasting for five hours, and worsening on arrival. There was no accompanying chest pain, back pain, or neurological deficits. He was a slender man with long fingers and extremities; his height was 185 cm (6' 1″) and weight was 70 kg. His blood pressure was 90/48 mmHg, compared to his normal systolic pressure of 110 mmHg. Oxygen saturation was 98% on room air. Head computed tomography (CT) showed no intracranial hemorrhage. Blood test revealed leukocytosis (19.8 x10^3/μL), an elevated creatinine (1.55 mg/dL), and an elevated D-dimer (31.9 µg/mL). Both the pain and blood pressure did not respond to 1000 mg acetaminophen infusion and 500 mL fluid infusion, and the point-of-care ultrasound of the inferior vena cava showed euvolemia. Given concern for persistently low blood pressure, we initiated telemetry, which showed ST depressions in lead II. The echocardiogram showed normal ejection fraction, dilated Valsalva sinus, severe aortic regurgitation, and an aortic flap. The electrocardiogram showed ST elevations in aVR and ST depressions in the other leads (Figure 1).

**Figure 2:**
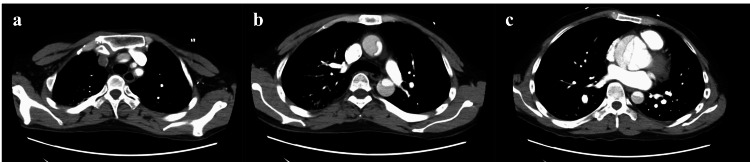
Contrast-enhanced computed tomography angiography of the chest (a) Dissection involving left common carotid artery, left vertebral artery, and brachiocephalic artery; (b) Dissection of ascending aorta and descending aorta with false cavity unstained; (c) Severely dilated Valsalva sinus with contrast entering a false cavity

Enhanced CT revealed a dissection from the Valsalva sinus to the level of the common iliac artery with extravasation of contrast from the aortic root. The patient was diagnosed with SAAD, with suspected entry from the aortic root (Figure 2).

**Figure 3:**
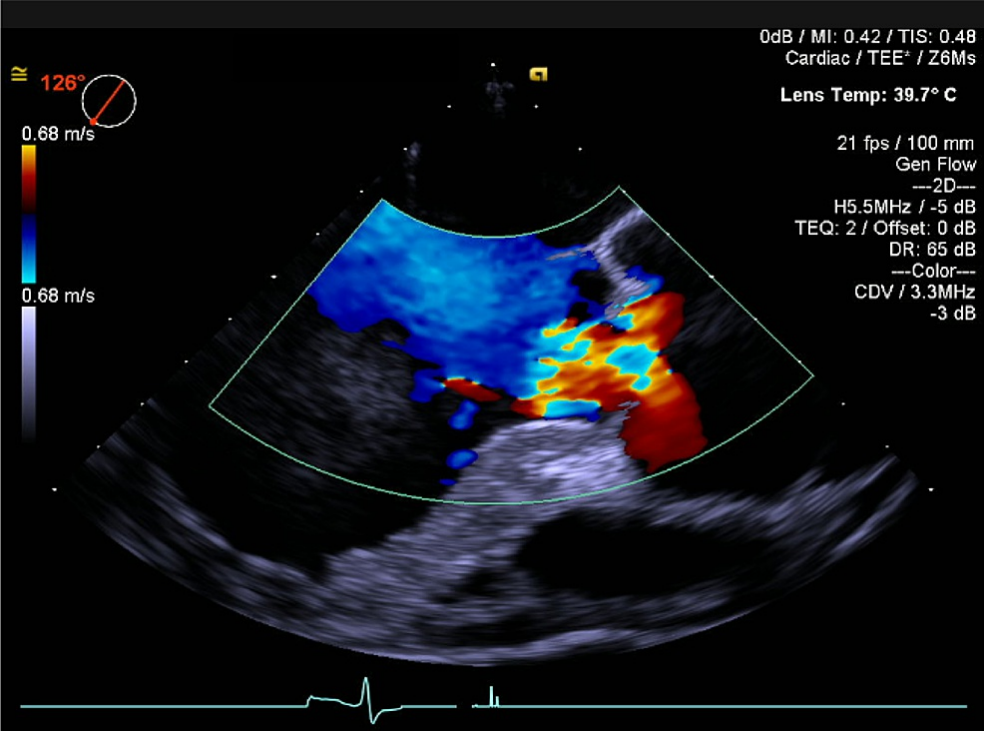
Transesophageal echocardiography during surgery

He was sent to the theater where severe aortic regurgitation was confirmed on a transesophageal echocardiogram, and he subsequently underwent aortic root replacement (Figure 3).

The entry of the intimal tear was found 2 cm above the non-coronary cusp. The patient was discharged from the hospital on postoperative day 24. At the one-year follow-up, the patient remained healthy and continued to work.

## Discussion

Chest pain that has a ripping or sharp quality and focal neurological deficits are classified as class I high-risk features of aortic dissection [12]. Compared to previously reported cases, this case is unusual in that it did not present with intense chest pain nor focal neurological symptoms, which decreased suspicion for aortic dissection (Table 1). The aortic dissection risk score was 0 points on arrival [13]. At the same time, the patient’s headache met the criteria for migraine, lasting for several hours with transient aura, as prescribed by The International Classification of Headache Disorders 3rd edition (ICHD-3) [14].

**Table 1:** Summary of published reports of patients with aortic dissection visited with migraine symptoms

Year/Author (Ref.)	Age/Sex	Range of aortic dissection	Migraine Symptoms	Chest pain	Neurological symptom
1998/Stollberger (11)	61/F	Sinus of Valsalva to aortic bifurcation	Bifrontal with nausea vomiting 1 hour prior to chest pain	Respiratory dependant right-sided pain	None
2004/Mathys (10)	53/M	Sinus of Valsalva to descending aorta	Parietotemporal / occipital headache 36 hours prior to chest pain, bifrontal headache 2 minutes after	Stabbing anterior chest pain for 10 minutes	None
2012/Seidel (9)	31/M	Ascending aorta to left subclavian artery	Severe bilateral facial pain and scotoma	None	Hypesthesia of upper limb
2021/Aoki (this case)	35/M	Sinus of Valsalva to aortic bifurcation	Diffuse frontal throbbing pain with aura	None	None

Furthermore, the patient described his headache and general malaise as being the “same as usual,” and mentioned drinking less water than normal due to the headache. Thus, we attributed his headache to migraine and suspected that volume depletion had caused his elevated creatinine and low blood pressure. Despite the presence of other explanations for his presentation, the actual diagnosis was aortic dissection complicated by severe aortic valve regurgitation and bilateral carotid artery dissection, which assumingly caused the headache (Figure 2).

Retrospectively, he had a positive wrist/thumb sign, pectus carinatum, dural ectasia, reduced upper/lower segment and increased arm/height ratio, and mild thoracolumbar kyphosis fulfilling an aortic dissection plus systemic score of 9 points [15]. The patient refused genetic testing for Marfan syndrome.

Marfan syndrome is believed to have a high prevalence of migraine possibly secondary to dural ectasia, as was present in this patient [16-17]. The effect of the physical anomalies associated with Marfan syndrome should not be overlooked. One possible etiology for the absence of chest pain in this patient could be the cystic medial necrosis of large vessels commonly observed in Marfan syndrome [18]. Chronically affected nerve bundles in the adventitia may have obscured the pain.

We must not underestimate the “regular migraine patient” at first glance. If vital signs are abnormal, one should consider an ultrasonography workup not only for volume status but also for cardiovascular findings, especially in the setting of Marfan-like features. 

## Conclusions

Stanford type A aortic dissection is one of the most fatal diseases that we encounter in emergency department settings. Our case of aortic dissection reminds us that devastating cardiovascular diseases may present with symptoms limited to migraine. Electrocardiogram and transthoracic echocardiogram may be considered for migraine patients, especially those with features of Marfan syndrome.
